# Dietary Fermented Strawberry Pomace Improves Yolk Lipid Quality and Modulates Reproductive Endocrine Activity, Intestinal Barrier Status, and Cecal Microbiota in Laying Hens

**DOI:** 10.3390/foods15183331

**Published:** 2026-09-20

**Authors:** Ting Chen, Binghua Qin, Md. Abul Kalam Azad, Siyu Yi, Zhihua Li, Yadong Cui, Xiangfeng Kong, Wei Lan

**Affiliations:** 1 Hunan Provincial Key Laboratory of Animal Nutritional Physiology and Metabolic Process, National Engineering Laboratory for Pollution Control and Waste Utilization in Livestock and Poultry Production, Institute of Subtropical Agriculture, Chinese Academy of Sciences, Changsha 410125, China; chenting23@mails.ucas.ac.cn (T.C.); qinbinghua23@mails.ucas.ac.cn (B.Q.); azadmak@isa.ac.cn (M.A.K.A.); siyuyi1996@163.com (S.Y.); zhli92@126.com (Z.L.); 2 College of Advanced Agricultural Sciences, University of Chinese Academy of Sciences, Beijing 100049, China; 3 School of Biology and Food Engineering, Fuyang Normal University, Fuyang 236037, China; fysyswx@163.com

**Keywords:** egg nutritional quality, fermented strawberry pomace, fruit processing by-product, gut barrier, laying hens, yolk fatty acids

## Abstract

Strawberry pomace is a fruit processing by-product rich in fiber and bioactive compounds, making it a functional feed resource. This study evaluated the effects of fermented strawberry pomace (FSP) on laying performance, yolk fatty acid composition, ovarian function, intestinal barrier status, cecal microbiota, and short-chain fatty acids (SCFAs) in laying hens. A total of 320 laying hens (345-day-old) were assigned to diets containing 0, 0.25, 0.5, or 1.0% FSP for 8 weeks. Supplementing 0.25% FSP increased albumen height and Haugh unit (*p* < 0.05). In addition, 1.0% FSP reduced yolk saturated fatty acids and thrombogenic index and increased the polyunsaturated-to-saturated fatty acid ratio (*p* < 0.05). Dietary FSP supplementation enhanced reproductive endocrine activity by increasing plasma and ovarian hormones, whereas 0.25% FSP upregulated *HSD17B1* and *LHCGR* expressions (*p* < 0.05). Dietary 1.0% FSP upregulated ovarian *Nrf2* and *SOD1* expressions, whereas 0.5% FSP improved small-intestinal morphology and upregulated intestinal barrier-associated gene expressions, including *Occludin*, *Cadherin 1*, and *Mucin 2* (*p* < 0.05). Moreover, 0.5% FSP increased cecal microbial richness, reshaped microbial composition, and 0.25–1.0% FSP supplementation increased the molar proportion of acetic acid but decreased absolute acetic acid and total SCFAs concentrations (*p* < 0.05). In summary, dietary FSP improved yolk lipid nutritional quality, particularly at 1.0% supplementation. FSP supplementation also affected reproductive endocrine activity, intestinal barrier-related status, and cecal microbial fermentation.

## 1. Introduction

Eggs are widely consumed animal-derived foods that provide high-quality protein, essential amino acids, lipids, vitamins, and minerals [1]. Beyond their contribution to dietary protein supply, the lipid composition of egg yolk has attracted increasing attention because it directly determines the nutritional and health-related values of eggs [2]. Notably, reducing the content of saturated fatty acids (SFAs) and increasing unsaturated fatty acids (UFAs), especially polyunsaturated fatty acids (PUFAs), are considered potential strategies for improving the nutritional value of egg yolk. From the human nutrition perspective, a higher PUFA/SFA ratio and more favorable lipid health indices, such as lower atherogenic and thrombogenic indices, are generally associated with a healthier fatty acid profile [3]. Therefore, improving the fatty acid composition of yolk represents an important strategy for enhancing the nutritional value of eggs.

Researchers have applied different nutritional strategies to improve the fatty acid composition of egg yolk. Previous studies have shown that the inclusion of lipid-rich feed ingredients, such as flaxseed, linseed oil, fish oil, and other plant-derived lipid sources, can alter yolk fatty acid deposition and enrich the content of UFAs in eggs [4,5,6]. In addition, agro-industrial by-products rich in bioactive compounds have been applied as alternative feed ingredients to improve egg quality and yolk lipid composition [7]. These strategies indicate that nutritional regulation is an effective way to enhance the functional value of eggs. However, conventional lipid-modifying strategies also have several limitations. For example, some lipid-rich ingredients may reduce feed palatability, impair production performance at higher inclusion levels, or increase yolk lipid susceptibility to oxidation due to their high degree of unsaturation [8]. Moreover, dietary fish oil or higher levels of docosahexaenoic acid supplementation have been shown to affect egg sensory quality by increasing fishy aroma and flavor, thereby limiting consumer acceptance [5]. Therefore, there is increasing interest in identifying sustainable feed additives that can improve yolk lipid quality while maintaining laying performance and oxidative stability.

Fruit pomaces are promising functional feed resources because they contain dietary fiber, residual lipids, polyphenols, organic acids, vitamins, and other bioactive compounds [9]. Using fruit pomaces in poultry diets may also support sustainable valorization of by-products and reduce competition for feed resources. Strawberry pomace, a major by-product generated during strawberry juice, jam, and puree production, contains polyphenols, such as anthocyanins, ellagic acid, quercetin, and catechins, as well as vitamins and other antioxidant constituents [10]. These compounds may regulate oxidative status, intestinal function, and lipid metabolism [9,11,12]. Notably, fermented strawberry pomace (FSP) may enhance yolk lipid deposition through multiple potential pathways, including the provision of residual UFAs and bioactive compounds, as well as improvements in antioxidant status and intestinal function [13]. Additionally, modulation of ovarian endocrine activity may further contribute to hepatic yolk-precursor synthesis, fatty acid transport, and subsequent lipid deposition in the egg yolk [12]. However, unprocessed strawberry pomace has several practical limitations, including high moisture content, poor storage stability, susceptibility to microbial contamination, and lower nutritional value than fresh strawberries [14]. These factors restrict its direct application as a feed additive.

Microbial fermentation is an effective strategy to improve the nutritional value of fruit by-products. Fermentation improves pomace stability, promotes the biotransformation of phytochemicals, increases the availability of bioactive compounds, improves protein and fiber utilization, and reduces anti-nutritional factors [15]. More importantly, fermented pomace is not only a nutrient source but also a multifunctional additive that can modulate antioxidant defense, intestinal barrier function, and gut microbiota. Previous studies have reported that fermented fruit pomaces improve egg quality, ovarian function, intestinal morphology, and microbial composition in laying hens [16]. Our previous research also revealed that FSP improved carcass characteristics and meat quality in laying hens during the late laying period by regulating plasma biochemical indices and antioxidant capacity [13]. These findings suggest that dietary FSP may have potential value in improving the productive and nutritional quality of laying hens.

Limited information is available regarding whether dietary FSP can improve egg quality in laying hens. We therefore hypothesized that dietary FSP supplementation might improve egg quality, particularly yolk lipid nutritional value, by modulating endocrine function, antioxidant capacity, and intestinal function in laying hens. Accordingly, this study evaluated the effects of dietary FSP supplementation on laying performance, egg quality, yolk fatty acid profile, ovarian function, intestinal morphology, barrier-related gene expression, cecal microbiota, and short-chain fatty acids (SCFAs) in laying hens.

## 2. Materials and Methods

### 2.1. Ethical Statement

All animal procedures were approved by the Institutional Animal Care and Use Committee of the Institute of Subtropical Agriculture, Chinese Academy of Sciences (approval no. ISA-2022-056; approved on 15 March 2022) and were conducted in accordance with the Regulations for the Administration of Affairs Concerning Experimental Animals (China).

### 2.2. Preparation and Fermentation of Strawberry Pomace

The fresh strawberry pomace was obtained from Fuyang Fruit Wine Engineering Technology Center (Fuyang, China). FSP was obtained by solid-state fermentation using a combined starter. Briefly, dried strawberry pomace was fermented for 64 h at 75 °C with 3–10% dissolved oxygen and 45–55% relative humidity. The combined starter consisted of microbial agents, active enzymes, culture medium 1 (artificially cultivated ginseng leaves), culture medium 2 (soybean powder), and sucrose at a mass ratio of 1:1:1:1:1. The microbial agents contained *Bacillus subtilis* (GDMCC 1.182), *Bacillus licheniformis* (GDMCC 1.648), *Lactobacillus plantarum* (GDMCC 2.215), and marine red yeast. The combined starter was mixed with strawberry pomace at a mass ratio of 1:100. The nutritional composition of the resulting FSP has been reported in our previous study [13]. The FSP contained 9.52% crude protein, 5.30% ether extract, 21.30% crude fiber, 11.20% crude ash, and 16.45 MJ/kg gross energy. Its fatty acid profile was dominated by linoleic acid (C18:2n-6, 42.53% of total fatty acids), oleic acid (C18:1n-9, 29.67%), palmitic acid (C16:0, 13.88%), α-linolenic acid (C18:3n-3, 8.68%), and stearic acid (C18:0, 3.09%).

### 2.3. Birds, Study Design, and Dietary Management

After seven days of adaptation, a total of 320 healthy 345-day-old Yukou Jingfen No. 8 laying hens were randomly assigned to four dietary treatments, consisting of a basal diet as the control and three diets obtained by adding 0.25%, 0.5%, or 1.0% FSP to the basal diet. Each treatment comprised eight replicates with 10 hens per replicate; each replicate contained two cages with five hens per cage receiving the same dietary treatment. Each replicate was treated as one experimental unit. Cages were arranged sequentially according to dietary treatment. The basal diet was designed to meet the nutrient recommendations of the national layer standard (NY/T 336-2004) (Supplementary Table S1). The experimental phase spanned eight weeks.

All birds originated from the same commercial flock and were selected based on similar body condition and laying performance at the beginning of the trial. All hens were kept in wire-floored battery cages, each equipped with a water nipple drinker and a feeder. The experimental house was maintained under controlled environmental conditions, including an ambient temperature of 18–24 °C, relative humidity of 45–60%, and a 16 h light/8 h dark cycle. To avoid additional handling stress that could transiently affect egg production, hens were not individually weighed before or during the trial. Throughout the trial, all hens had free access to drinking water and feed. An earlier non-peer-reviewed version of this work based on the same experimental cohort was posted on SSRN [17].

### 2.4. Data Collection and Sampling

The number and total weight of eggs per replicate were recorded daily to determine the egg laying rate (ELR) and average egg weight (AEW). Feed intake and leftovers were recorded weekly to estimate average daily feed intake (ADFI) and the feed-to-egg ratio (FER).

At 2, 4, 6, and 8 weeks of the trial, three eggs per replicate (24 eggs per treatment) were randomly collected for egg quality analysis. Egg quality measurements were averaged within the replicate, and the replicate (*n* = 8) was used as the experimental unit for statistical analysis. At the end of the feeding trial (week 8), one hen was selected from each replicate within each treatment, resulting in eight sampled hens per treatment. Thus, each sampled hen represented one independent experimental replicate for terminal blood and tissue analyses. Blood samples (5 mL) were drawn from the brachial wing vein into EDTA-coated anticoagulant tubes. Afterward, blood samples were centrifuged at 3000× *g* for 10 min at 4 °C, and the resulting plasma samples were immediately frozen at −20 °C until further analysis of reproductive hormone levels. After euthanasia by cervical dislocation, the ovaries were excised for evaluation of follicle number, antioxidant capacity, and reproductive hormone levels. Segments of the mid-jejunum and mid-ileum were collected and divided into two portions. One portion was fixed in 4% paraformaldehyde for morphological examination, whereas the other portion was immediately snap-frozen in liquid nitrogen and stored at −80 °C until RNA extraction. For intestinal morphology analysis, four of the eight sampled hens per treatment were randomly selected (*n* = 4), with each hen originating from a different replicate of the same treatment group. Cecal contents (*n* = 8) were aseptically collected immediately after dissection, transferred into sterile cryogenic tubes, snap-frozen in liquid nitrogen, and stored at −80 °C until microbiota and SCFAs analyses.

### 2.5. Determination of Egg Quality

Eggs collected at 2, 4, 6, and 8 weeks of the trial were used to assess the Haugh unit, albumen height, and yolk color with an Egg Analyzer (Orka Food Technology Ltd., Ramat Hasharon, Israel). Eggshell thickness was measured at the top, middle, and bottom of the eggs without the shell membrane with a hand-held micrometer (Deli Group, Ningbo, China). The mean values of the three readings were used to calculate the eggshell thickness. Additionally, eggshell strength was assessed using an eggshell strength tester (Orka Food Technology Ltd., Ramat Hasharon, Israel). The egg shape index was calculated from the horizontal and vertical diameters measured with a Vernier Caliper (Shanghai Shenhan Measures Co., Ltd., Shanghai, China). The eggshell, yolk, and albumen percentages were calculated using the following formulas:

Eggshell percent (%) = eggshell weight (g)/total egg weight (g) × 100

Yolk percent (%) = yolk weight (g)/total egg weight (g) × 100

Albumen percent (%) = (total egg weight − yolk weight − eggshell weight) (g)/total egg weight (g) × 100

### 2.6. Determination of Egg Nutritional Value

At 8 weeks of the trial, one egg was randomly selected from six different replicates per treatment (*n* = 6) for evaluation of crude protein and amino acid content in egg albumen, as well as crude fat and fatty acid compositions in egg yolk. The crude protein content in egg albumen was determined using a KT200 Kjeldahl analyzer (Foss Analytical Co., Ltd., Suzhou, China) following the Chinese National Standard GB5009.5-2016 [18]. The amino acid content in egg albumen was determined with an L-8800 Amino Acid Analyzer (Hitachi Ltd., Tokyo, Japan) according to the standard protocols established by GB5009.124-2016 [19]. Crude fat content in egg yolk was evaluated in accordance with the Chinese guideline GB 5009.6-2016 [20]. Fatty acids in egg yolk were profiled by gas chromatography (Nexis GC-2030 system; Shimadzu Corporation, Kyoto, Japan) in accordance with GB 5009.168-2016 [21].

### 2.7. Assessment of Follicle Development

The ovarian follicles in the ovary of laying hens were categorized into four groups based on size and appearance, including large white follicles (LWF, diameter 3–5 mm), small yellow follicles (SYF, diameter 5–8 mm), hierarchy follicles (HF, diameter > 12 mm), and large yellow follicles (LYF, diameter 8–12 mm). Follicle counts were calculated using the previously described methods [22].

### 2.8. Ovarian Oxidative Status and Antioxidant Capacity

The levels of malondialdehyde (MDA), glutathione (GSH), glutathione peroxidase (GSH-Px), superoxide dismutase (SOD), and total antioxidant capacity (T-AOC) in the ovary were determined with ELISA kits from the Nanjing Jiancheng Bioengineering Institute Ltd. (Nanjing, China). These indices were measured on a Microplate Reader Infinite M200 PRO (Tecan, Männedorf, Switzerland) in accordance with the manufacturer’s protocols.

### 2.9. Determination of Reproductive Hormones

The levels of follicle-stimulating hormone (FSH), luteinizing hormone (LH), and estradiol (E_2_) in plasma and ovary were measured using ELISA kits purchased from Hunan Ruicheng Biotechnology Co., Ltd. (Changsha, China). These assays were performed on a Microplate Reader Infinite M200 PRO (Tecan, Männedorf, Switzerland) following the manufacturers’ protocols.

### 2.10. Intestinal Morphology Analysis

Jejunal and ileal tissues preserved in 4% paraformaldehyde were fixed, dehydrated, xylene-cleared, and then processed for paraffin embedding. A 5-μm section of the embedded tissues was prepared and stained with hematoxylin–eosin (H&E). Villus width (VW), villus height (VH), and crypt depth (CD) were measured under a light microscope (Nikon ECLIPSE 80i, Tokyo, Japan). The villus surface area (VSA) and VH-to-CD ratio (VCR) were calculated according to the following equations:

$$VSA=\pi \times \frac{VW}{2}\times \sqrt{\left[{\left(\frac{VW}{2}\right)}^{2}+VH^{2}\right]}$$

VCR = VH/CD

### 2.11. Gene Expression Analysis

The total RNA from the jejunum, ileum, and ovary was isolated using AG RNAex Pro (Accurate Biology, Changsha, China) in accordance with the manufacturer’s protocols. The concentration and purity of the isolated RNA were assessed with a Nanophotometer N60 (Implen GmbH, Munich, Germany). Consequently, the purified RNA was then reverse-transcribed to cDNA using the Evo M-MLV Reverse Transcription Assay Kit (Accurate Biology, Changsha, China). SYBR Green Premix Pro Taq HS (Accurate Biology, Changsha, China) was used for real-time PCR, and reactions were run on a LightCycler 480 II instrument (Roche Applied Science, Basel, Switzerland) to determine gene expression levels. Each qPCR reaction (10 μL) contained 1 μL cDNA template, 0.4 μL of forward primer, 0.4 μL of reverse primer, 5 μL SYBR Green Premix (Accurate Biology, Changsha, China), and 3.2 μL nuclease-free water. The qPCR program consisted of an initial denaturation at 95 °C for 5 min, followed by 45 cycles of denaturation at 95 °C for 10 s, annealing at 53 °C for 10 s, and extension at 72 °C for 20 s. A melting curve analysis was performed at the end of amplification to verify the specificity of PCR products. Relative expression levels of the target genes were calculated using the 2^−ΔΔCT^ value [23]. *β-Actin* was selected as the reference gene based on its previous use in laying hens under similar experimental conditions [16]. The Ct values of *β-actin* did not differ significantly among dietary treatments (*p* > 0.05, Supplementary Table S3), supporting its stability under the present experimental conditions. The specific primer sequences for all primers are provided in Supplementary Table S2.

### 2.12. Cecal Microbiota Analysis

Sequencing and analysis of the cecal microbiota were performed by Shanghai Personal Biotechnology Co., Ltd. (Shanghai, China). Total microbial DNA was isolated from cecal digesta, and the V3–V4 hypervariable region of the bacterial 16S rRNA gene was amplified using universal primer pairs, with an amplicon size of approximately 468 bp. The primer sequences were as follows: forward, 5′-ACTCCTACGGGAGGCAGCA-3′; reverse, 5′-GGACTACHVGGGTWTCTAAT-3′. The resulting PCR amplicons were purified before sequencing and subjected to paired-end sequencing on an Illumina NovaSeq system (Illumina, San Diego, CA, USA). A total of 3,986,814 forward reads (R1) were obtained from the 32 samples, with a mean sequencing depth of 124,588 reads per sample. QIIME2 (version 2019.4) was used to process the sequences, and DADA2 plugin was used for sequence denoising and inference of amplicon sequence variants (ASVs). Taxonomic assignment of ASVs was performed against the Greengenes database (Release 13.8). Alpha-diversity indices were evaluated to characterize microbial richness and diversity. Beta diversity was evaluated using NMDS based on Bray–Curtis dissimilarity, with group differences assessed by ANOSIM. Differences in taxonomic abundance at the genus level were assessed using the Kruskal–Wallis test, with *p* values adjusted using the Benjamini–Hochberg FDR procedure. PICRUSt2 (version 2.2.0) was used to predict microbial functional potential, and predicted KEGG pathways were summarized at level 3.

### 2.13. Cecal Short-Chain Fatty Acids Analysis

Approximately 0.5 g of cecal digesta was mixed with 2.5 mL of double-distilled water and stored overnight at 4 °C. The mixture was then centrifuged at 10,000× *g* for 10 min to obtain the supernatant. The supernatant was diluted with 2 mL of double-distilled water and incubated with thorough mixing for 30 min. After centrifugation under the same conditions, the resulting supernatant was transferred to a 5 mL colorimetric tube. Following the addition of 25% metaphosphoric acid, the sample was centrifuged again at 12,000× *g* and 4 °C for 15 min. The obtained supernatant was filtered through a 0.45-µm membrane and subsequently injected into a 7890A gas chromatograph (Agilent, Santa Clara, CA, USA) for the determination of SCFAs [24].

### 2.14. Statistical Analysis

Statistical analyses were performed using SPSS Statistics 26.0 (IBM Corp., Armonk, NY, USA). Data normality and homogeneity of variance were assessed using the Shapiro–Wilk and Levene’s tests, respectively. Production performance and egg-quality traits were analyzed using a mixed model, with dietary treatment, time, and their interaction as fixed effects. Model parameters were estimated using restricted maximum likelihood (REML), and estimated marginal means were compared using the least significant difference (LSD) procedure when significant effects were detected. All other variables were analyzed using one-way ANOVA followed by Tukey’s post hoc test. Data are presented as means ± SEM. Differences were considered significant at *p* < 0.05, and trends were considered at 0.05 ≤ *p* < 0.10.

## 3. Results

### 3.1. Production Performance

The effects of dietary FSP on production performance of laying hens are summarized in Table 1. The repeated-measures mixed-model analysis showed no significant effects of dietary treatment or treatment × time interaction on ELR, AEW, ADFI, or FER (*p* > 0.05). A significant effect of time was observed for ADFI, with ADFI being highest during weeks 5–6 and lowest during weeks 3–4 (*p* < 0.001). Time also affected FER, which was higher during weeks 5–6 than during weeks 1–2 and 3–4 (*p* < 0.05).

### 3.2. Egg Quality

The results of egg quality traits are presented in Table 2. Dietary treatment significantly affected egg albumen height and Haugh unit (*p* < 0.05), with no significant treatment × time interactions for these traits (*p* > 0.05). The 0.25% FSP group had higher albumen height and Haugh unit than the control and 1.0% FSP groups (*p* < 0.05). Significant treatment × time interactions were observed for albumen percent (*p* < 0.05) and eggshell percent (*p* < 0.001), and these interactions were further examined by simple-effects analysis (Table 3). At week 2 of the trial, the 1.0% FSP group had a higher egg albumen percent and a lower eggshell percent compared with the 0.25% and 0.5% FSP groups (*p* < 0.05). At week 4 of the trial, a tendency for a treatment effect on eggshell percentage was observed (*p* = 0.065), with the highest value in the 0.5% FSP group.

### 3.3. Egg Nutritional Value

Egg nutritional values, including ether extract, fatty acids, and health lipid indices in egg yolk, are presented in Table 4. The C17:0 content was higher in the 1.0% FSP group than in the other groups (*p* < 0.05) and showed a significant quadratic response to increasing dietary FSP level (*p* < 0.05). Compared with the control group, the C20:3n6 content was lower in the 0.25% and 0.5% FSP groups (*p* < 0.05), and overall C20:3n6 content showed a significant linear dose–response to dietary FSP level (*p* < 0.05). Additionally, the C18:2n6c content displayed a tendency toward higher values in hens receiving 1.0% FSP than in the other three groups (*p* = 0.087).

The proportion of SFAs and the thrombogenic index (TI) decreased linearly (*p* < 0.05), while the ratio of PUFA/SFA was higher in the 1.0% FSP group, when compared with the control group (*p* < 0.05). Notably, the n-6 PUFA (*p* = 0.098) content, as well as the ratio of hypocholesterolemia to hypercholesterolemia (H/H; *p* = 0.066) and health-promoting index (HPI, *p* = 0.081) displayed an increasing trend with increasing dietary FSP level. In addition, the atherosclerosis index (AI) displayed a decreasing trend with increasing dietary FSP level (*p* = 0.065).

Crude protein and amino acid profiles in egg albumen are shown in Supplementary Table S4. Proline content was lower (*p* < 0.05) in the 0.25% and 1.0% FSP groups relative to the control group. However, no significant treatment effects were observed for crude protein or other amino acid content in egg albumen among groups (*p* > 0.05).

### 3.4. Reproductive Hormones in Plasma and Ovary

The concentrations of reproductive hormones in plasma and ovary are presented in Table 5. Plasma FSH concentration was higher (*p* < 0.05) in the 0.25% and 0.5% FSP groups relative to the control and 1.0% FSP groups, whereas plasma LH was higher (*p* < 0.05) in the 0.25% and 1.0% FSP groups than in the 0.5% FSP and control groups. Moreover, ovarian FSH, LH, and E_2_ levels were linearly increased (*p* < 0.05) in all FSP-supplemented groups compared to the control group.

### 3.5. Number of Ovarian Follicles and Expression of Genes Involved in Hormonal Biosynthesis and Receptor Signaling

**Figure 1:**
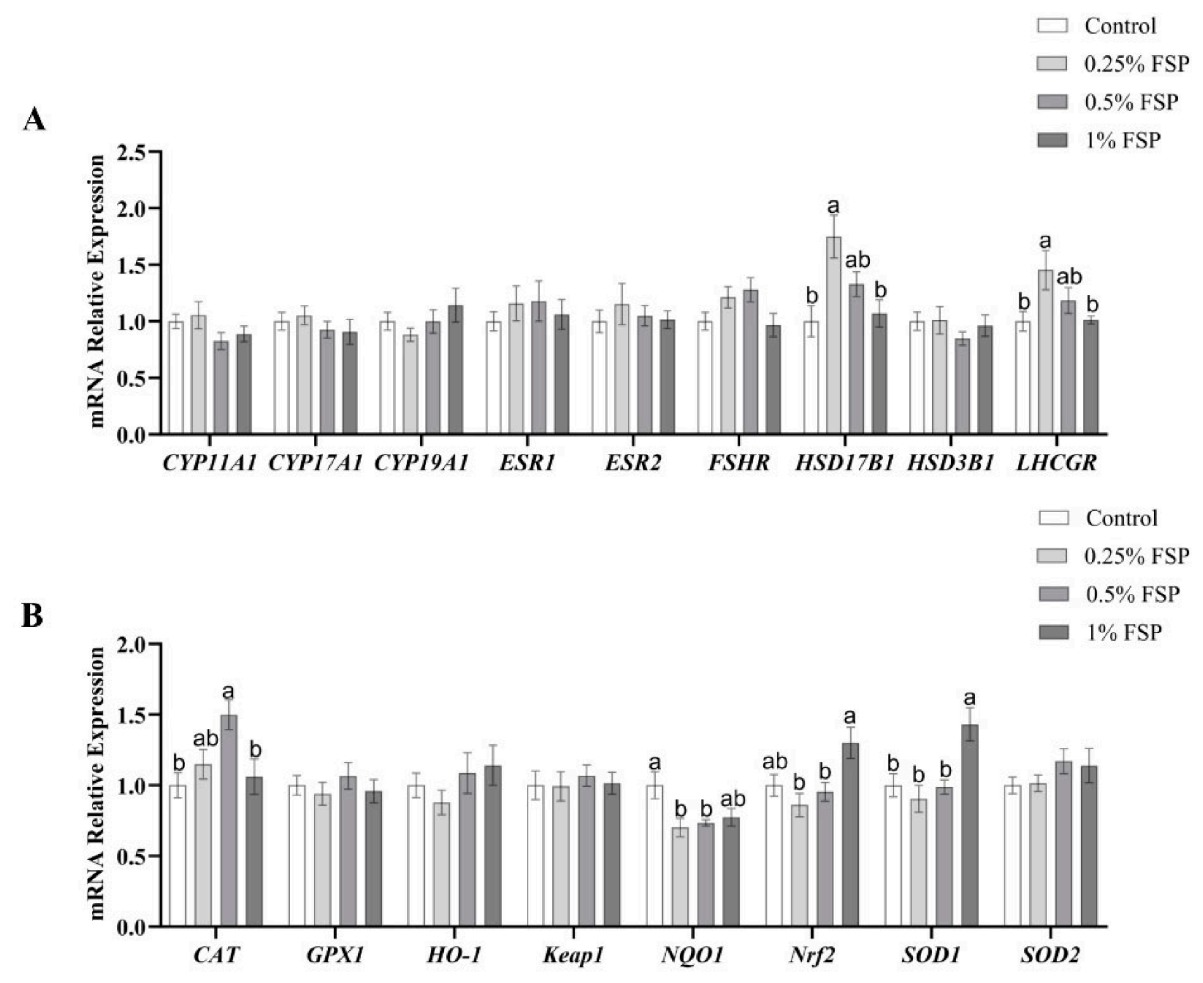
Effects of dietary fermented strawberry pomace (FSP) supplementation on ovarian hormone synthesis, receptor expression (**A**), and antioxidant-related gene expression (**B**) in laying hens. Data are expressed as means with SEM as error bars (*n* = 8). Bars with different lowercase letters differ significantly (*p* < 0.05). *CYP11A1*, cytochrome P450 family 11 subfamily A member 1; *CYP17A1*, cytochrome P450 family 17 subfamily A member 1; *CYP19A1*, cytochrome P450 family 19 subfamily A member 1; *ESR1*, estrogen receptor 1; *ESR2*, estrogen receptor 2; *FSHR*, follicle stimulating hormone receptor; *HSD17B1*, hydroxysteroid 17-beta dehydrogenase 1; *HSD3B1*, hydroxysteroid 3-beta dehydrogenase 1; *LHCGR*, luteinizing hormone/choriogonadotropin receptor; *CAT*, catalase; *HO-1*, heme oxygenase 1; *GPX1*, glutathione peroxidase 1; *Keap1*, Kelch-like ECH-associated protein 1; *NQO1*, NAD(P)H quinone dehydrogenase 1; *Nrf2*, nuclear factor erythroid 2-related factor 2; *SOD1*, superoxide dismutase 1; *SOD2*, superoxide dismutase 2.

There were no significant differences (*p* > 0.05) in ovarian follicle count among the groups (Table 5). As shown in Figure 1A, the hydroxysteroid 17-beta dehydrogenase 1 (*HSD17B1*) and luteinizing hormone/choriogonadotropin receptor (*LHCGR*) expressions were upregulated (*p* < 0.05) in the ovary of hens fed 0.25% FSP relative to the control and 1.0% FSP groups.

### 3.6. Ovarian Antioxidant Capacity and Related Gene Expressions

The antioxidant status in the ovary of laying hens is presented in Table 5. Hens receiving 0.25% FSP showed lower ovarian GSH-Px activity than the remaining three groups (*p* < 0.05). However, dietary FSP had no significant effects (*p* > 0.05) on T-AOC, MDA, SOD, GSH, and CAT levels in the ovary of hens.

As shown in Figure 1B, dietary 1.0% FSP upregulated (*p* < 0.05) ovarian *Nrf2* expression compared with the 0.25 and 0.5% FSP groups, as well as ovarian *SOD1* expression compared with other groups. Additionally, ovarian *NQO1* expression was downregulated (*p* < 0.05) in the 0.25% and 0.5% FSP groups compared with the control group. Moreover, ovarian *CAT* expression showed an upregulation (*p* < 0.05) in the 0.5% FSP group relative to the 1.0% FSP and control groups.

### 3.7. Intestinal Morphology

**Figure 2:**
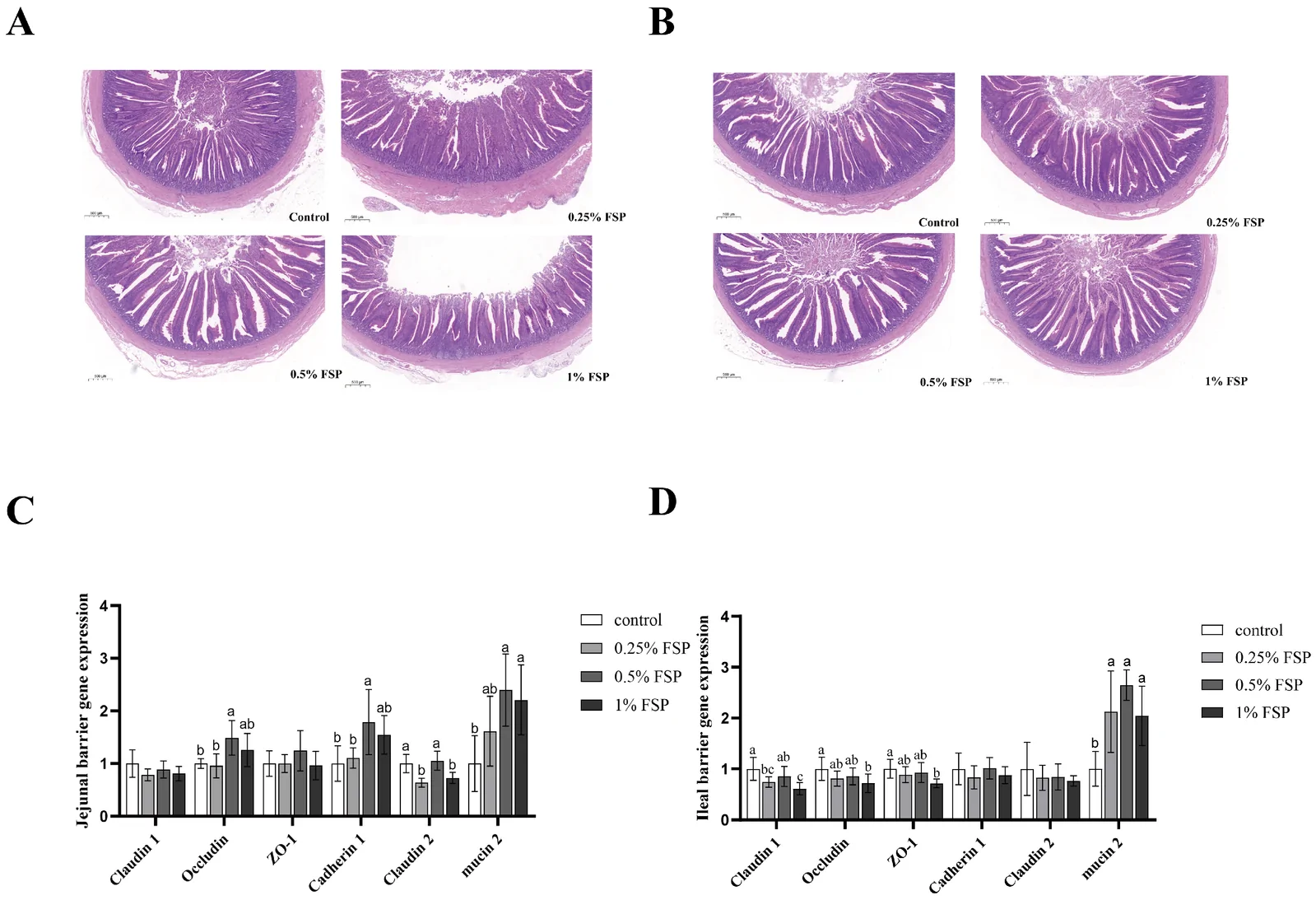
Effects of dietary fermented strawberry pomace (FSP) supplementation on intestinal morphology (*n* = 4) and barrier-related gene expression (*n* = 8) in laying hens. Representative histological sections of jejunum and ileum, respectively, stained with H&E. Scale bars, 500 μm (magnification 20×) (**A**,**B**). Relative mRNA expression levels of jejunal barrier genes and ileal barrier genes, respectively (**C**,**D**). Data are expressed as means with SEM as error bars. Bars with different lowercase letters differ significantly (*p* < 0.05).

Intestinal morphology results are presented in Figure 2A,B and Table 6. The H&E staining of intestinal tissues shows a well-preserved villus architecture in all FSP-supplemented groups, with structural compactness in comparison with the control group (Figure 2A,B). The VW and VSA in the jejunum were higher in the 0.5% FSP group compared with the control group (*p* < 0.05). In the ileum, VH was higher in the 0.5% FSP group than in the control group (*p* = 0.050), and VSA was higher in the 1.0% FSP group than in the control group (*p* < 0.05; Table 6).

### 3.8. Expression of Intestinal Barrier-Related Genes

The expression levels of intestinal barrier-related genes are illustrated in Figure 2C,D. In the jejunum, *Occludin* and *Cadherin 1* expression were upregulated (*p* < 0.01) in the 0.5% FSP group compared with the control and 0.25% FSP groups, as well as *Mucin 2* expression in the 0.5 and 1.0% FSP groups compared with the control group. In addition, the *Claudin 2* expression was downregulated (*p* < 0.05) in the 0.25 and 1.0% FSP groups compared with the control and 0.5% FSP groups. Moreover, the *Claudin 1* expression displayed a downregulating trend (*p* = 0.085) in all FSP-supplemented groups compared to the control group (Figure 2C). In the ileum, *Mucin 2* expression was upregulated (*p* < 0.05) in all FSP-treated groups than in the control group. However, the *Claudin 1* expression was downregulated (*p* < 0.05) in the 0.25% and 1.0% FSP groups when compared with the control group. In addition, the *Occludin* and *ZO-1* expressions were downregulated (*p* < 0.05) in the 1.0% FSP group compared with the control group (Figure 2D).

### 3.9. Cecal Microbiota Structure, Functional Potential, and Short-Chain Fatty Acids

**Figure 3:**
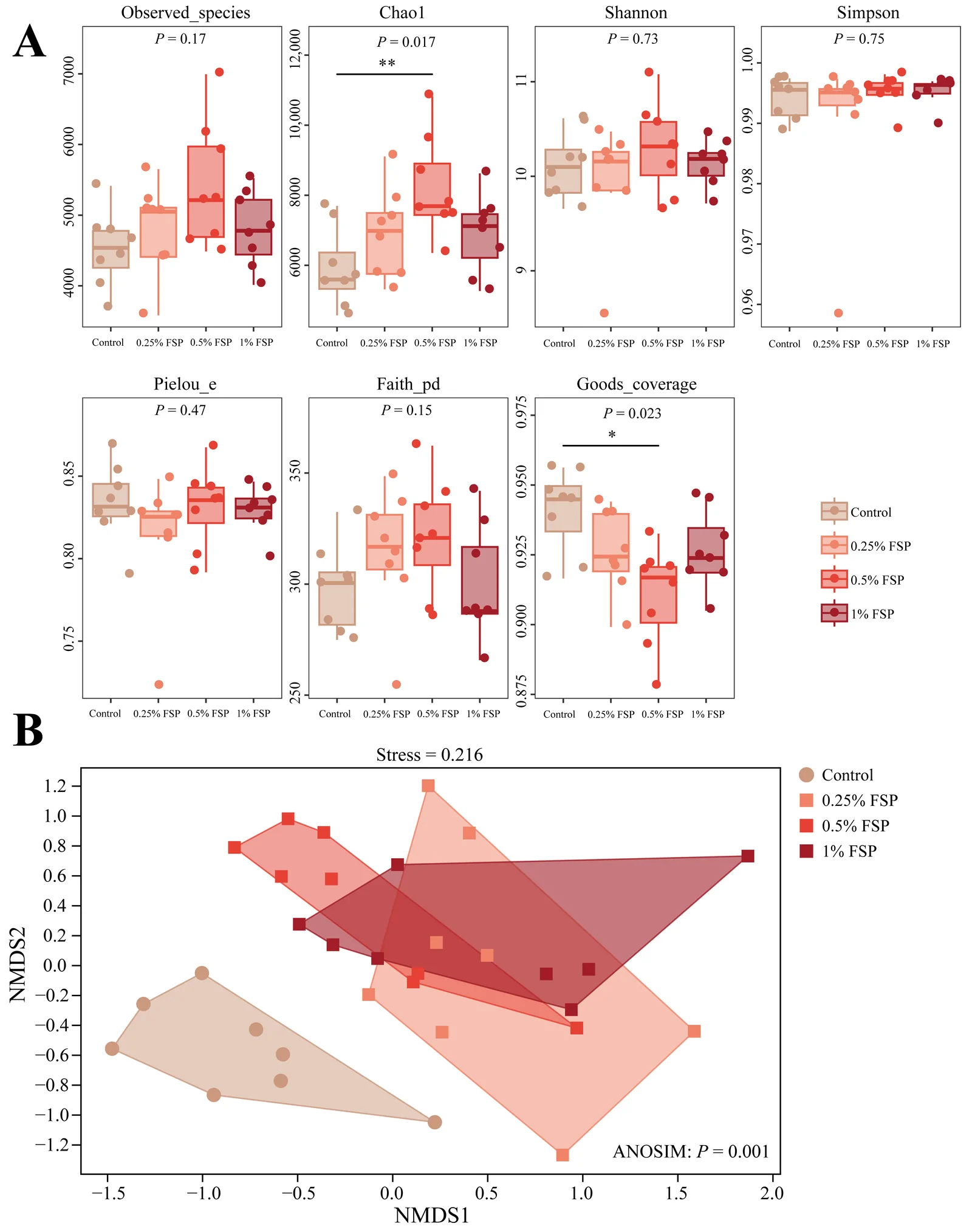
Effects of dietary fermented strawberry pomace (FSP) supplementation on cecal microbiota (**A**) α-diversity and (**B**) β-diversity in laying hens (*n* = 8). * *p* < 0.05, ** *p* < 0.01.

**Figure 4:**
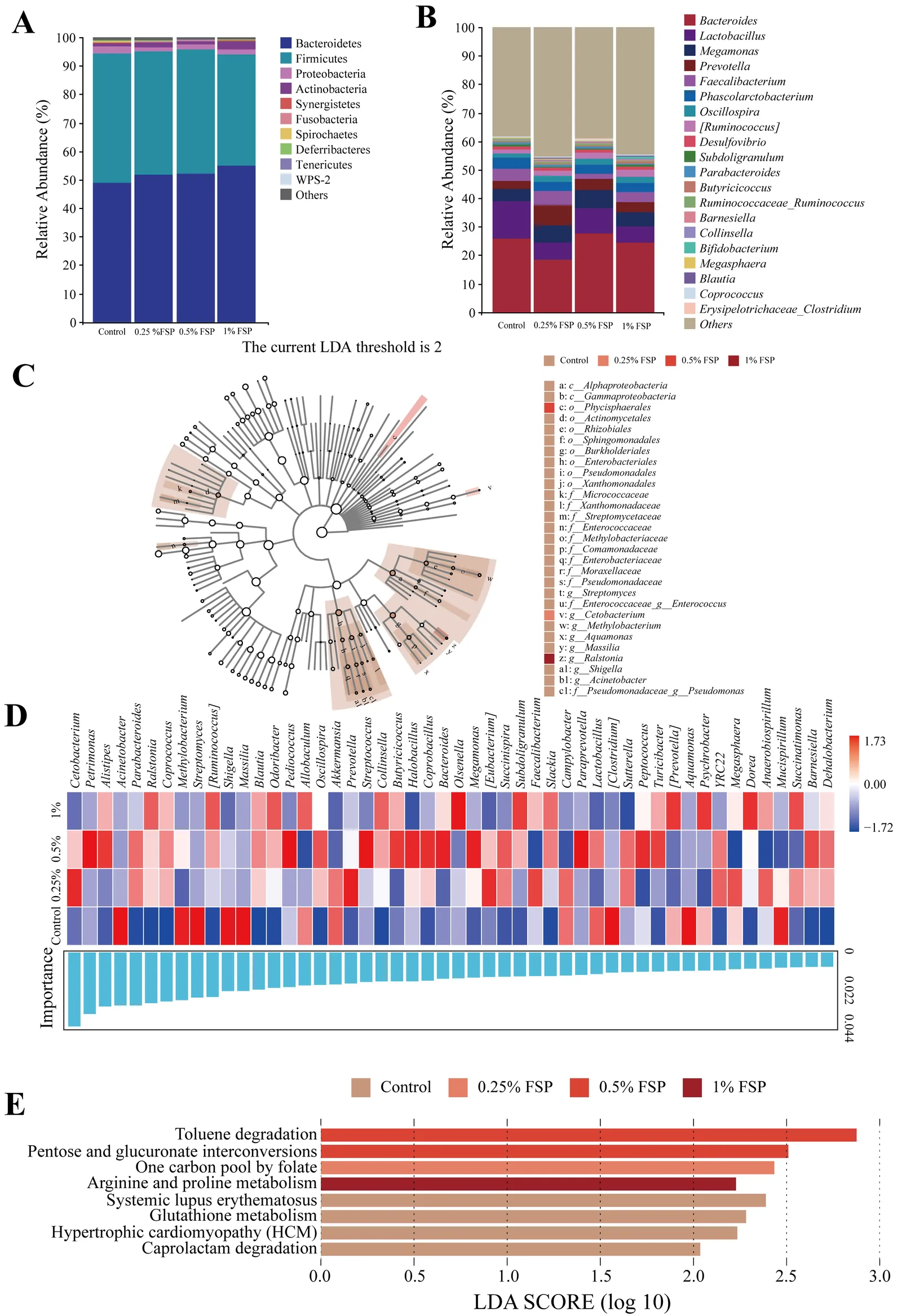
Cecal microbiome responses to dietary fermented strawberry pomace (FSP) supplementation in laying hens: microbial composition, differential taxa, and predicted functional potential (*n* = 8). (**A**) Phylum-level relative abundance; (**B**) genus-level relative abundance; (**C**) LEfSe cladogram; (**D**) random forest analysis; and (**E**) PICRUSt2-predicted KEGG pathways.

The α-diversity indices of cecal microbiota are shown in Figure 3A. The 0.5% FSP treatment group showed an increased Chao 1 index (*p* < 0.05), but a decreased Good’s_coverage index (*p* < 0.05) compared with the control group. As illustrated in Figure 3B, NMDS score plots of the FSP-supplemented groups show distinct clustering of cecal microbiota from the control group. The structure of the cecal microbiota at the phylum and genus levels, as well as the differential genera, is displayed in Figure 4. At the phylum level (Figure 4A), Bacteroidetes, Firmicutes, Proteobacteria, and Actinobacteria were the most dominant phyla across all groups. At the genus level (Figure 4B), *Bacteroides* and *Lactobacillus* represented the two most dominant genera. Furthermore, dietary FSP supplementation increased the relative abundances of *Ruminococcus*, *Ruminococcaceae_Ruminococcus*, *Blautia*, and *Coprococcus* compared with the control group.

To identify the genera contributing to differences in cecal microbial communities among treatments, LEfSe analysis was performed at the genus level using an LDA score threshold of ≥2. *Cetobacterium* was enriched in the 0.25% FSP group, whereas *Ralstonia* was enriched in the 1.0% FSP group (Figure 4C). Random forest analysis further identified *Cetobacterium*, *Petrimonas*, *Alistipes*, *Acinetobacter*, and *Parabacteroides* as the major genera contributing to treatment discrimination (Figure 4D). Predicted microbial functional profiles were further elucidated using PICRUSt2 at KEGG level 3 (Figure 4E). The one-carbon pool by the folate pathway was predicted to be enriched in the 0.25% FSP group. Pathways related to toluene degradation and pentose and glucuronate interconversions were predicted to be enriched in the 0.5% FSP group, whereas arginine and proline metabolism were predicted to be enriched in the 1.0% FSP group.

The results of cecal SCFAs are presented in Table 7. Compared with the control group, 0.25–1.0% FSP supplementation decreased the total SCFA, acetate, propionate, butyrate, and valerate concentrations (*p* < 0.05). In terms of molar percentage, 0.25–1.0% FSP increased the acetate percent and decreased the propionate and butyrate percentages (*p* < 0.05), whereas the valerate percent was not affected (*p* > 0.05).

**Figure 5:**
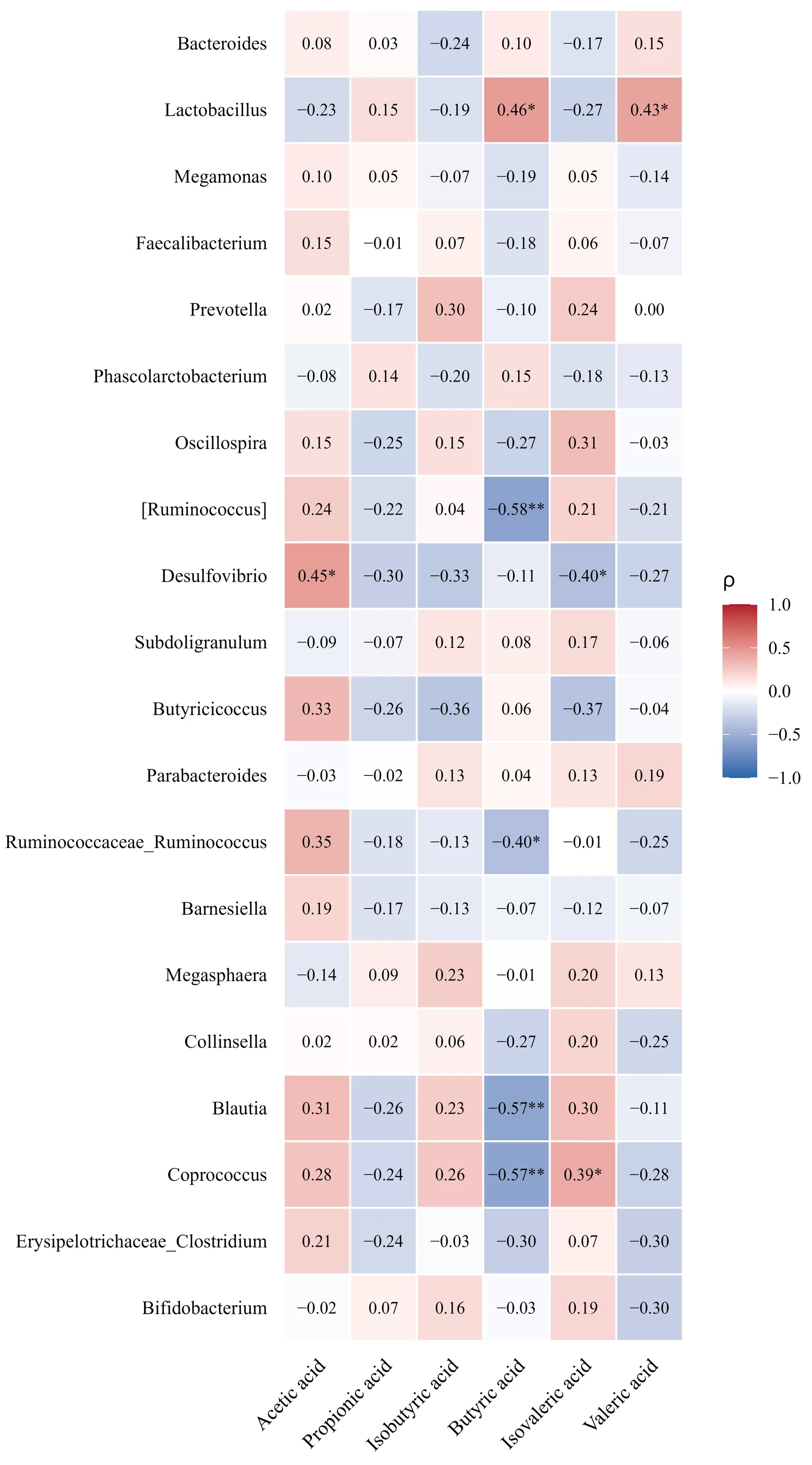
Correlation analysis between cecal short-chain fatty acids molar percentages and microbiota abundance at genus level. Red represents a significant positive correlation, and blue represents a significant negative correlation. * *p* < 0.05, ** *p* < 0.01.

Correlation analysis revealed that the acetate molar percentage was significantly and positively correlated with *Desulfovibrio* (ρ = 0.45, *p* < 0.05; Figure 5). Although [*Ruminococcus*], *Ruminococcaceae_Ruminococcus*, *Blautia*, and *Coprococcus* showed positive numerical associations with acetate molar percentage, these correlations were not statistically significant. Notably, these genera were negatively associated with butyrate molar percentage, including [*Ruminococcus*] (ρ = −0.58, *p* < 0.01), *Ruminococcaceae_Ruminococcus* (ρ = −0.40, *p* < 0.05), *Blautia* (ρ = −0.57, *p* < 0.01), and *Coprococcus* (ρ = −0.57, *p* < 0.01).

## 4. Discussion

Egg quality and yolk lipid composition are closely associated with ovarian endocrine activity, intestinal function, and gut microbial metabolism in laying hens. Therefore, nutritional strategies targeting these physiological processes may provide an effective approach for improving egg quality and yolk lipid nutritional value. In the present study, dietary 0.25% FSP increased egg albumen height and Haugh unit. Dietary 1.0% FSP improved the yolk fatty acid profile, as indicated by a reduced SFA proportion and TI and an increased PUFA/SFA ratio. These changes were accompanied by alterations in reproductive hormone levels, intestinal morphology, barrier-related gene expression, cecal microbial composition, and SCFA profiles, although the causal relationships among these factors require further investigation.

As a key nutritional indicator, feed intake is closely associated with the production performance and egg quality of laying hens. In the present study, dietary FSP supplementation did not significantly affect overall ELR, AEW, ADFI, and FER, indicating that the inclusion of FSP at 0.25–1.0% had no adverse effect on fed intake and laying performance. Previous studies have reported that dietary supplementation with fermented blueberry pomace and fermented *Aronia melanocarpa* pomace both improved the ADFI of hens without consistently impairing laying performance [12,25]. However, a similar treatment effect on feed intake was not observed in the present study. The ADFI was significantly affected by time. Thus, the temporal variation in ADFI appeared to reflect changes over the course of the trial rather than a specific effect of FSP supplementation.

Albumen is composed of various proteins, peptides, and amino acids, and the Haugh unit indicates the freshness of eggs, which are important indicator related to egg quality [26]. In the present study, the 0.25% FSP group had increased albumen height and Haugh unit. In addition, a significant treatment × time interaction was observed for albumen percent, with the 1.0% FSP group showing a higher albumen percent than the 0.25% and 0.5% FSP groups at week 2. In contrast, the amino acid composition of albumen was generally unaffected by FSP supplementation, except for the proline content. Consistent with the present study, Reis et al. [11] found that layers supplemented with 1.0–3.0% grape pomace flour for 35 days under heat stress had higher albumen pH and Haugh unit (after storage) without affecting albumen percentage. Previous studies have also shown that E_2_ regulates the development and secretory activity of the oviduct magnum and promotes albumen protein synthesis [27]. In the present study, FSP supplementation increased ovarian E_2_ level, which was accompanied by changes in egg albumen height and Haugh unit. Therefore, these responses may be associated with the concurrent changes in ovarian E_2_.

From the nutritional perspective, a lower percentage of SFA and a higher PUFA/SFA ratio are generally regarded as favorable features of the fatty acid profile [28,29]. In the present study, compared with the control group, supplementation of 1.0% FSP reduced SFA percentage and increased PUFA/SFA ratio in yolk. These findings are in agreement with a previous study that reported that dietary inclusion of 2–6% dried olive pulp in laying hens’ diet increased the percentage of PUFA, decreased the percentage of SFA, and improved the PUFA/SFA ratio in eggs [30]. Therefore, the present results indicate that dietary FSP, particularly at 1.0% dose, altered the yolk fatty acid profile toward a more favorable nutritional composition. Moreover, compared with the control group, 1.0% FSP increased the percentage of C17:0 and tended to increase that of C18:2n6c in the yolk. These changes indicate alteration in specific components of the yolk fatty acid profile. C17:0 and C18:2n6c have been reported as fatty acids relevant to nutritional lipid assessment [31,32]. Therefore, dietary FSP supplementation appeared to modulate specific yolk fatty acids toward a potentially more favorable nutritional profile. The fatty acid composition of egg yolk is strongly influenced by the lipid source and fatty acid composition of the diets [33]. Lan et al. [13] reported that the FSP also had higher percentages of PUFA (51.21%) and C18:2n6c (42.53%), which may inhibit the synthesis of SFA and alter the fatty acid composition in yolk. Therefore, supplementing 1.0% FSP optimized the composition of fatty acids in the yolk, which may be due to the rich fatty acids in FSP.

The AI, TI, HPI, H/H, and desired fatty acid (DFA) are calculated indices commonly used to characterize the nutritional quality of the fatty acid profile. Lower AI and TI values, together with higher HPI, H/H, and DFA values, generally indicate a more favorable fatty acid profile [3]. In the present study, the 1.0% FSP group had a lower TI value and tended to decrease the AI value compared with the control group. Moreover, dietary FSP (0.25–1.0%) supplementation also tended to increase the H/H and HPI values in the yolk. Overall, these findings demonstrate that dietary FSP improved the fatty acid profile of egg yolk, with the most pronounced changes observed at the 1.0% FSP supplementation level.

Oxidative stress accelerates ovarian aging by inducing apoptosis, inflammation, and mitochondrial dysfunction [34]. In the present study, dietary 1.0% FSP upregulated ovarian *SOD1* expression, while *CAT* expression was also upregulated in the 0.5% FSP group compared with the control group. Moreover, ovarian *NQO1* expression was downregulated in the 0.25% and 0.5% FSP groups compared with the control group. Additionally, ovarian GSH-Px activity was lower in the 0.25% FSP group compared with the other groups. Taken together, these results indicate that FSP supplementation altered ovarian antioxidant-related gene expressions, particularly at the 1.0% supplementation level. However, these transcriptional responses were not consistently accompanied by corresponding changes in antioxidant enzyme activities. Therefore, whether FSP improves functional ovarian antioxidant defense remains unknown. Further dose–response studies integrating antioxidant-related gene expression and enzyme activities are needed to determine the optimal FSP supplementation level.

The secretion of reproductive hormones in laying hens is associated with the deposition of fatty acids in egg yolk. Among reproductive hormones, E_2_ has the strongest mechanistic link with yolk lipid deposition through regulating hepatic yolk precursor synthesis and long-chain PUFA biosynthesis [35]. Furthermore, FSH and LH are also likely to affect yolk lipid deposition indirectly by modulating follicular development and ovarian steroidogenesis [36]. In the present study, ovarian FSH, LH, and E_2_ levels were increased in laying hens supplemented with FSP. Furthermore, plasma FSH content was significantly higher in the 0.25% and 0.5% FSP groups, and plasma LH content was higher in the 0.25% and 1.0% FSP groups than in the control group. The fermentation of pomace can enrich several bioactive compounds, such as organic acids, phenolics, and prebiotic components [37], which may exert beneficial effects on reproductive function in poultry. For example, supplementation with fermented *Aronia melanocarpa* pomace has been reported to enhance ovarian FSH, LH, and E_2_ levels in laying hens, further supporting the beneficial effects of fermented pomace on reproductive function and production-related traits [12]. Previous studies have reported that LHCGR is localized in the theca and granulosa cells of ovarian follicles and plays a crucial role in E_2_ production [38]. HSD17B1 is mainly expressed in the placenta and ovarian granulosa cells and efficiently converts the less active E_1_ into the highly active E_2_ [39]. In the present study, ovarian *LHCGR* and *HSD17B1* expressions were upregulated in the 0.25% FSP group, suggesting altered ovarian responsiveness and estrogenic steroid metabolism. Considering the established involvement of E_2_ in hepatic yolk precursor synthesis and fatty acid metabolism, these endocrine and ovarian steroidogenic responses may be associated with yolk lipid deposition. However, the mechanistic link between these responses and the altered yolk fatty acid profile remains to be clarified.

The morphological structure of the intestine reflects its functional capacity and health status. In the present study, dietary 0.5% FSP supplementation enhanced small-intestinal structure, as evidenced by increased VW and VSA in the jejunum and increased VH in the ileum. Moreover, dietary 1.0% FSP supplementation further increased ileal VSA compared with the control group. The development of intestinal morphology is found to be related to feed intake [40], and better intestinal morphology often indicates the expansion of absorption surface and the enhancement in nutrient absorption [41]. Therefore, the morphological changes observed in the present study may reflect an increased absorptive capacity of the small intestine.

Furthermore, dietary FSP altered the expression of intestinal barrier-associated genes, and these effects varied with dietary FSP level. In the jejunum, 0.5% FSP upregulated *Occludin* and *Cadherin 1* expression, and 0.25% and 1.0% FSP downregulated *Claudin 2* expression compared with the control group, which may reflect alterations in the expression of genes related to junctional integrity and paracellular permeability [42,43,44]. Additionally, *Mucin 2* expression was upregulated in the jejunum of the 0.5% and 1.0% FSP groups, as well as upregulated in the ileum across all FSP-supplemented groups. These findings suggest alterations in mucus barrier-related gene expression throughout the small intestine [45]. In the ileum, however, higher FSP levels produced less favorable or adaptive responses, with *Claudin 1* downregulation in the 0.25% and 1.0% FSP groups, while *Occludin* and *ZO-1* were downregulated in the 1.0% FSP group, indicating that barrier-related gene expression responses were not uniformly favorable at higher FSP levels [46]. Overall, the intestinal barrier-related responses to FSP were dose-dependent. The 0.5% FSP group exhibited a more favorable pattern of intestinal morphology and jejunal barrier-related gene expression, together with increased *Mucin 2* expression in both the jejunum and ileum.

The SCFAs are mainly produced by microbial fermentation of carbohydrates in the cecum of laying hens. The production and absorption of SCFAs are in a dynamic equilibrium state to maintain the pH balance in the intestine [47]. The absorption of SCFAs is mainly mediated by surface cells through molecular mechanisms, which are closely related to intestinal morphology and transporters [47,48]. In the present study, dietary FSP supplementation decreased total SCFA concentration and the absolute concentrations of acetate, propionic acid, and butyric acid, while increasing the molar percentage of acetate and decreasing the molar percentages of propionate and butyrate. This pattern suggests that dietary FSP reshaped the relative cecal fermentation profile, with acetate accounting for a larger proportion of the total SCFA pool. Acetate absorbed in the intestine can be converted into acetyl-CoA in the liver and participate in de novo fatty acid synthesis [49,50,51]. However, in the present study, the absolute concentration of cecal acetate decreased despite an increase in its molar percentage. Therefore, the observed change should be interpreted as an alteration in the relative SCFA profile rather than evidence of increased acetate production or hepatic acetate availability. However, whether cecal acetate metabolism contributes to yolk fatty acid deposition requires further investigation.

The increased molar percentage of acetate and decreased molar percentages of propionate and butyrate may be partly associated with the alteration in acetate-associated bacterial genera and overall microbial fermentation patterns. In the present study, *Ruminococcus*, *Ruminococcaceae_Ruminococcus*, *Blautia*, and *Coprococcus* were positively correlated with the molar percentage of acetate and negatively correlated with the molar percentage of butyrate. Moreover, *Desulfovibrio* was also positively correlated with the molar percentage of acetic acid. *Ruminococcus* was generally considered an acetogenic bacterium, which mainly produces more acetate rather than propionate and butyrate by decomposing cellulose in the intestine [52]. *Blautia* was also found to be the main acetate-producing bacterium [53,54], and *Coprococcus* and *Desulfovibrio* mainly produce acetate in the intestine [55,56,57]. In the present study, dietary 0.25–1.0% FSP supplementation increased the relative abundances of *Ruminococcus*, *Ruminococcaceae_Ruminococcus, Blautia*, and *Coprococcus*, which coincided with the increased molar percentage of acetate with dietary FSP supplementation. However, the relative abundance of *Desulfovibrio* was not significantly affected by FSP supplementation. Further studies are needed to determine how these microbial changes relate to the observed shift in the relative SCFA profile, because acetate production was not directly measured and the absolute acetate concentration decreased following FSP supplementation. Therefore, dietary FSP supplementation altered the cecal microbial composition and SCFA molar profile, particularly by increasing the relative contribution of acetate. These microbial and fermentation-related changes may be associated with the improvement in yolk fatty acid composition, but their causal relationship requires further investigation.

## 5. Conclusions

In summary, dietary FSP improved yolk lipid nutritional quality in laying hens, as indicated by a reduced SFA proportion and TI and an increased PUFA/SFA ratio, with 1.0% FSP showing the most pronounced effect. Dietary FSP supplementation was also associated with changes in reproductive endocrine activity, enhanced intestinal morphology, altered barrier-related gene expression, and shifts in cecal microbiota and SCFA profiles, particularly an increased acetate molar percentage. Therefore, 1.0% FSP may have potential for improving yolk lipid quality in laying hens. However, the present study still has several limitations, including the absence of an unfermented strawberry pomace control, analysis of the fatty acid composition of the final diets, and direct evidence linking the observed reproductive endocrine, intestinal, microbial, and SCFA changes with yolk fatty acid deposition. Further studies are needed to clarify these relationships and underlying mechanisms and to validate the effects of FSP under practical production conditions and over longer feeding periods.

## Figures and Tables

**Table 1:** Effects of dietary fermented strawberry pomace (FSP) supplementation on production performance of laying hens.

Item	Control	Dietary FSP Level (%)			Time (Week)				SEM	*p*-Values		
		0.25	0.5	1.0	1–2	3–4	5–6	7–8		Treatment	Time	Treatment × Time
ELR (%)	71.58	71.92	69.96	72.92	72.70	69.22	71.70	72.77	1.259	0.415	0.164	0.666
AEW (g)	51.58	51.48	51.87	51.43	51.58	51.58	51.47	51.73	0.248	0.599	0.910	0.209
ADFI (g)	96.29	96.94	97.04	97.19	96.83 ^C^	90.64 ^D^	101.48 ^A^	98.51 ^B^	0.333	0.242	<0.001	0.172
FER	2.62	2.66	2.70	2.61	2.60 ^B^	2.58 ^B^	2.77 ^A^	2.65 ^AB^	0.049	0.534	0.033	0.585

Data are presented as means with their SEM (*n* = 8). Different uppercase superscripts letters within the Time columns indicate significant differences among time periods (*p* < 0.05). ELR, egg laying rate; AEW, average egg weight; ADFI, average daily feed intake; FER, feed-to-egg ratio.

**Table 2:** Effects of dietary fermented strawberry pomace (FSP) supplementation on egg quality of laying hens.

Item	Control	Dietary FSP Level (%)			Time (Week)				SEM	*p*-Values		
		0.25	0.5	1.0	2	4	6	8		Treatment	Time	Treatment × Time
Eggshell strength (N/m^2^)	39.85	39.81	40.00	40.95	40.30	39.69	40.96	39.66	0.777	0.698	0.600	0.748
Egg shape index	1.334	1.331	1.332	1.322	1.320	1.327	1.337	1.334	0.005	0.402	0.116	0.284
Albumen height (mm)	4.32 ^b^	4.93 ^a^	4.58 ^ab^	4.47 ^b^	3.84 ^C^	4.63 ^B^	4.77 ^AB^	5.06 ^A^	0.130	0.011	<0.001	0.189
Yolk color	13.64	13.69	13.79	13.81	13.57 ^B^	13.25 ^B^	14.03 ^A^	14.07 ^A^	0.125	0.736	<0.001	0.534
Haugh unit	63.49 ^b^	69.61 ^a^	66.82 ^ab^	65.34 ^b^	60.73 ^C^	66.25 ^B^	68.08 ^AB^	70.20 ^A^	1.234	0.006	<0.001	0.498
Eggshell thickness (mm)	0.340	0.336	0.337	0.333	0.342 ^A^	0.331 ^B^	0.345 ^A^	0.329 ^B^	0.003	0.487	<0.001	0.390
Albumen percent (%)	55.45	54.63	55.11	55.36	52.80 ^B^	56.28 ^A^	55.68 ^A^	55.79 ^A^	0.300	0.222	<0.001	0.002
Eggshell percent (%)	13.19 ^b^	13.64 ^a^	13.58 ^a^	13.04 ^b^	13.91 ^A^	12.98 ^C^	13.20 ^BC^	13.36 ^B^	0.119	<0.001	<0.001	<0.001
Yolk percent (%)	31.35	31.78	31.30	31.58	33.31 ^A^	30.73 ^B^	31.12 ^B^	30.86 ^B^	0.265	0.549	<0.001	0.113

Data are presented as means with their SEM (*n* = 8). Different lowercase superscripts letters within the Treatment columns indicate significant differences among dietary treatments, whereas different uppercase superscripts letters within the Time columns indicate significant differences among time periods (*p* < 0.05).

**Table 3:** Effects of dietary fermented strawberry pomace (FSP) supplementation on albumen and eggshell percentages at different time points in laying hens.

Item (%)	Control	Dietary FSP Level (%)			SEM	*p*-Values
		0.25	0.5	1.0		
Week 2 of the trial						
Eggshell percent	13.34 ^ab^	14.87 ^a^	14.70 ^a^	12.74 ^b^	0.198	<0.001
Albumen percent	53.88 ^ab^	51.19 ^b^	51.29 ^b^	54.83 ^a^	0.468	0.004
Week 4 of the trial						
Eggshell percent	13.23	12.92	13.26	12.53	0.112	0.065
Albumen percent	56.66	56.29	56.26	55.92	0.229	0.750
Week 6 of the trial						
Eggshell percent	13.12	13.22	13.11	13.33	0.099	0.856
Albumen percent	55.54	55.38	56.60	55.20	0.289	0.323
Week 8 of the trial						
Eggshell percent	13.05	13.56	13.24	13.58	0.141	0.502
Albumen percent	55.70	55.67	56.28	55.49	0.256	0.738

Data are presented as means with their SEM (*n* = 8). Different lowercase superscripts letters within a row indicate statistically significant differences at *p* < 0.05.

**Table 4:** Effects of dietary fermented strawberry pomace (FSP) supplementation on ether extract content (g/100 g yolk), fatty acid percentage (%), and health-related lipid indices in egg yolk.

Item	Control	Dietary FSP Level (%)			SEM	*p*-Values
		0.25	0.5	1.0		
Ether extract	33.89	33.35	33.50	33.85	0.293	0.908
C14:0	0.40	0.38	0.38	0.38	0.009	0.777
C16:0	25.78	25.34	24.96	24.63	0.181	0.120
C16:1n7c	3.12	2.84	2.72	2.66	0.077	0.155
C17:0	0.14 ^b^	0.15 ^ab^	0.15 ^ab^	0.17 ^a^	0.005	0.018
C18:0	8.26	8.04	8.24	7.75	0.118	0.420
C18:1n9c	42.90	43.62	43.87	42.11	0.439	0.512
C18:2n6c	16.26	16.61	16.49	19.09	0.458	0.087
C18:3n3	0.65	0.67	0.65	0.75	0.004	0.384
C18:3n6	0.12	0.12	0.11	0.14	0.004	0.219
C20:1n9c	0.22	0.23	0.21	0.23	0.022	0.416
C20:2n6	0.13	0.13	0.12	0.15	0.004	0.273
C20:3n6	0.14 ^a^	0.11 ^b^	0.12 ^b^	0.13 ^ab^	0.003	0.010
C20:4n6	1.29	1.20	1.24	1.24	0.016	0.244
C22:6n3	0.60	0.56	0.57	0.57	0.010	0.573
Health lipid indice						
SFA (%)	34.58 ^a^	33.90 ^ab^	33.73 ^ab^	32.93 ^b^	0.194	0.016
MUFA (%)	46.23	46.69	46.80	45.00	0.443	0.482
PUFA (%)	19.19	19.40	19.30	22.07	0.495	0.108
PUFA/SFA	0.56 ^b^	0.57 ^ab^	0.57 ^ab^	0.67 ^a^	0.017	0.039
n-3 PUFA (%)	1.25	1.24	1.22	1.32	0.028	0.629
n-6 PUFA (%)	17.94	18.17	18.08	20.75	0.473	0.098
n-6/n-3 PUFA	14.32	14.69	14.86	15.70	0.231	0.187
AI	0.42	0.41	0.40	0.39	0.004	0.065
TI	0.96 ^a^	0.93 ^ab^	0.93 ^ab^	0.89 ^b^	0.008	0.017
H/H	2.36	2.44	2.48	2.56	0.028	0.066
HPI	2.39	2.46	2.50	2.58	0.026	0.081
DFA (%)	73.68	74.13	74.35	74.82	0.181	0.157

Data are presented as means with their SEM (*n* = 6). Different lowercase superscripts letters within a row indicate statistically significant differences at *p* < 0.05. SFA, saturated fatty acid; PUFA, polyunsaturated fatty acid; MUFA, monounsaturated fatty acid; TI, thrombogenic index; AI, atherogenic index; H/H, hypocholesterolemia/hypercholesterolemia; HPI, health promoting index; DFA, desired fatty acid. TI = (C14:0 + C16:0 + C18:0)/(0.5 × MUFA + 0.5 × n-6 PUFA + 3 × n-3 PUFA + n-3/n-6 PUFA); AI = (4 × C14:0 + C16:0)/UFA; HH ratio = (C18:1n-9 + C18:2n-6 + C20:4n-6 + C18:3n-3 + C20:5n-3 + C22:5n-3 + C22:6n-3)/(C14:0 + C16:0); HPI = (MUFA + PUFA)/(C12:0 + 4 × C14:0 + C16:0); DFA = C18:0 + MUFA + PUFA.

**Table 5:** Effects of dietary fermented strawberry pomace (FSP) supplementation on follicle numbers, reproductive hormones, and ovarian antioxidant capacity of laying hens.

Item	Control	Dietary FSP Level (%)			SEM	*p*-Values
		0.25	0.5	1.0		
Follicle numbers						
LWF	20.00	26.63	21.25	24.63	1.165	0.162
HF	4.38	4.63	4.00	4.00	0.127	0.229
SYF	5.00	6.13	6.00	4.25	0.408	0.327
LYF	0.63	1.13	1.13	1.13	0.090	0.116
Plasma reproductive hormones						
FSH (mIU/mL)	25.29 ^b^	37.83 ^a^	33.87 ^a^	19.16 ^b^	1.623	<0.001
LH (mIU/mL)	12.20 ^b^	18.65 ^a^	14.57 ^b^	19.51 ^a^	0.730	<0.001
E_2_ (pmol/L)	67.82	67.57	62.61	58.73	1.929	0.290
Ovarian reproductive hormones						
FSH (mIU/mg prot)	2.97 ^b^	4.88 ^a^	4.95 ^a^	5.31 ^a^	0.273	0.005
LH (mIU/mg prot)	1.30 ^b^	3.55 ^a^	3.75 ^a^	3.48 ^a^	0.267	0.001
E_2_ (pmol/g prot)	9.12 ^b^	15.51 ^a^	15.14 ^a^	15.76 ^a^	0.775	0.002
Ovarian antioxidant capacity						
T-AOC (mM/mg)	0.24	0.21	0.28	0.28	0.014	0.191
MDA (nM/mg prot)	1.05	1.00	1.09	0.92	0.078	0.899
SOD (U/mL)	12.45	15.26	16.56	16.51	0.881	0.322
GSH (U/mg prot)	1.57	1.46	1.28	1.50	0.067	0.448
CAT (U/mg prot)	255.65	408.18	420.62	366.86	34.334	0.321
GSH-Px (U/mg prot)	8.57 ^a^	4.77 ^b^	10.91 ^a^	11.21 ^a^	0.940	0.049

Data are presented as means with their SEM (*n* = 8). Different lowercase superscripts letters within a row indicate statistically significant differences at *p* < 0.05. LWF, large white follicles (3–5 mm); SYF, small yellow follicle (5–8 mm); LYF, large yellow follicle (8–12 mm); HF, hierarchy follicle (>12 mm); FSH, follicle-stimulating hormone; LH, luteinizing hormone; E_2_, estradiol; T-AOC, total antioxidant capacity; MDA, malondialdehyde; SOD, superoxide dismutase; GSH, glutathione; CAT, catalase; GSH-Px, glutathione peroxidase.

**Table 6:** Effects of dietary fermented strawberry pomace (FSP) supplementation on intestinal morphology of laying hens.

Item	Control	Dietary FSP Level (%)			SEM	*p*-Values
		0.25	0.5	1.0		
jejunum						
CD (μm)	193.51	219.90	194.48	218.75	6.999	0.380
VH (μm)	1017.24	1235.53	1249.12	1081.58	46.092	0.203
VW (μm)	213.11 ^b^	253.43 ^ab^	302.98 ^a^	223.35 ^ab^	13.040	0.044
VCR	5.62	5.86	6.71	5.18	0.304	0.369
VSA (μm^2^)	344.11 ^b^	508.73 ^ab^	590.77 ^a^	383.78 ^ab^	34.962	0.025
ileum						
CD (μm)	152.17	153.33	187.38	168.82	9.336	0.549
VH (μm)	1012.59 ^b^	1098.24 ^ab^	1365.91 ^a^	1162.80 ^ab^	49.275	0.050
VW (μm)	207.59	193.21	263.46	333.55	21.937	0.077
VCR	7.13	7.82	7.87	7.21	0.324	0.814
VSA (μm^2^)	337.41 ^b^	323.82 ^b^	568.20 ^ab^	615.21 ^a^	48.044	0.034

Data are presented as means with their SEM (*n* = 4). Different lowercase superscripts letters within a row indicate statistically significant differences at *p* < 0.05. CD, crypt depth; VH, villus height; VW, villus width; VCR, VH-to-CD ratio; VSA, villus surface area.

**Table 7:** Effects of dietary fermented strawberry pomace (FSP) supplementation on cecal short-chain fatty acid (SCFA) content and molar percentage of laying hens.

Item	Control	Dietary FSP Level (%)			SEM	*p*-Values
		0.25	0.5	1.0		
Concentration (μmol/g)						
Acetic acid	74.90 ^a^	55.15 ^b^	60.25 ^b^	60.76 ^b^	2.128	0.001
Propionic acid	20.83 ^a^	12.51 ^b^	12.02 ^b^	13.19 ^b^	0.886	<0.001
Isobutyric acid	3.62 ^a^	3.19 ^ab^	2.63 ^b^	2.38 ^b^	0.172	0.046
Butyric acid	5.24 ^a^	2.82 ^b^	2.58 ^b^	2.46 ^b^	0.299	<0.001
Isovaleric acid	1.50	1.36	1.16	0.98	0.081	0.144
Valeric acid	3.08 ^a^	1.90 ^b^	2.07 ^b^	1.93 ^b^	0.175	0.029
Total SCFAs	109.16 ^a^	76.92 ^b^	80.70 ^b^	81.70 ^b^	3.420	<0.001
Molar percentage (%)						
Acetic acid	68.87 ^b^	71.74 ^a^	74.59 ^a^	74.41 ^a^	0.637	0.001
Propionic acid	18.94 ^a^	16.27 ^b^	14.89 ^b^	16.18 ^b^	0.400	<0.001
Isobutyric acid	3.30	4.19	3.31	2.95	0.184	0.092
Butyric acid	4.78 ^a^	3.58 ^b^	3.17 ^b^	2.86 ^b^	0.199	0.001
Isovaleric acid	1.35	1.80	1.46	1.24	0.089	0.125
Valeric acid	2.77	2.41	2.57	2.36	0.119	0.644

Data are presented as means with their SEM (*n* = 8). Different lowercase superscripts letter within a row indicates statistically significant differences at *p* < 0.05.

## Data Availability

The data presented in this study are available in the ScienceDB repository at https://www.scidb.cn/en/s/RVvmYb (accessed on 14 September 2026).

## Supplementary Materials

The following supporting information can be downloaded at: https://www.mdpi.com/article/10.3390/foods15183331/s1, Table S1. Ingredients and nutrient content of the basal diet (%, as feed basis). Table S2. Primer sequences used for quantitative real-time PCR analysis. Table S3. Ct values of β-actin in ovarian samples from different dietary treatments. Table S4. Effects of dietary fermented strawberry pomace supplementation on crude protein and amino acid composition of egg albumen in laying hens (g/100 g).

## References

[1] Virtanen J.K., Larsson S.C. (2024). Eggs—A scoping review for Nordic Nutrition Recommendations 2023. Food Nutr. Res..

[2] Milinsk M.C., Murakami A.E., Gomes S., Matsushita M., De Souza N.E. (2003). Fatty acid profile of egg yolk lipids from hens fed diets rich in n-3 fatty acids. Food Chem..

[3] Chen J., Liu H. (2020). Nutritional indices for assessing fatty acids: A mini-review. Int. J. Mol. Sci..

[4] Alagawany M., Elnesr S.S., Farag M.R., Abd El-Hack M.E., Khafaga A.F., Taha A.E., Tiwari R., Yatoo M.I., Bhatt P., Khurana S.K. (2019). Omega-3 and omega-6 fatty acids in poultry nutrition: Effect on production performance and health. Animals.

[5] Feng J., Long S., Zhang H., Wu S., Qi G., Wang J. (2020). Comparative effects of dietary microalgae oil and fish oil on fatty acid composition and sensory quality of table eggs. Poult. Sci..

[6] Vlaicu P.A., Panaite T.D., Turcu R.P. (2021). Enriching laying hens eggs by feeding diets with different fatty acid composition and antioxidants. Sci. Rep..

[7] Mnisi C.M., Mhlongo G., Manyeula F. (2022). Fruit pomaces as functional ingredients in poultry nutrition: A review. Front. Anim. Sci..

[8] Aziza A.E., Panda A.K., Quezada N., Cherian G. (2013). Nutrient digestibility, egg quality, and fatty acid composition of brown laying hens fed camelina or flaxseed meal. J. Appl. Poult. Res..

[9] Erinle T.J., Adewole D.I. (2022). Fruit pomaces-their nutrient and bioactive components, effects on growth and health of poultry species, and possible optimization techniques. Anim. Nutr..

[10] Basu A., Nguyen A., Betts N.M., Lyons T.J. (2014). Strawberry as a functional food: An evidence-based review. Crit. Rev. Food Sci. Nutr..

[11] Reis J.H., Gebert R.R., Barreta M., Boiago M.M., Souza C.F., Baldissera M.D., Santos I.D., Wagner R., Laporta L.V., Stefani L.M. (2019). Addition of grape pomace flour in the diet on laying hens in heat stress: Impacts on health and performance as well as the fatty acid profile and total antioxidant capacity in the egg. J. Therm. Biol..

[12] Li Z., Qin B., Chen T., Kong X., Zhu Q., Azad M., Cui Y., Lan W., He Q. (2024). Fermented *Aronia melanocarpa* pomace improves the nutritive value of eggs, enhances ovarian function, and reshapes microbiota abundance in aged laying hens. Front. Microbiol..

[13] Lan W., Chen T., Qin B., Azad M.A.K., Li Z., Cui Y., Kong X. (2025). Fermented strawberry pomace enhances carcass characteristics and meat quality by regulating plasma biochemical indices and antioxidant capacity in aged laying hens. Front. Nutr..

[14] Hotchkiss A.T., Chau H.K., Strahan G.D., Nunez A., Harron A., Simon S., White A.K., Dieng S., Heuberger E.R., Black I. (2024). Structural characterization of strawberry pomace. Heliyon.

[15] Hashimoto E.H., de Cassia Campos Pena A., Da Cunha M.A.A., de Freitas Branco R., de Lima K.P., Couto G.H., Binder Pagnoncelli M.G. (2025). Fermentation-mediated sustainable development and improvement of quality of plant-based foods: From waste to a new food. Syst. Microbiol. Biomanuf..

[16] Qin B., Li Z., Azad M., Chen T., Cui Y., Lan W., Wang H., Kong X. (2024). Fermented blueberry pomace supplementation improves egg quality, liver synthesis, and ovary antioxidant capacity of laying hens. Poult. Sci..

[17] Chen T., Qin B., Azad M.A.K., Li Z., Yi S., Lan W., Cui Y., Kong X. (2025). Dietary fermented strawberry pomace improves egg quality in aged laying hens by enhancing antioxidant capacity, strengthening the intestinal barrier function, and modulating the cecal microbiota. SSRN Electron. J..

[18] (2016). National Food Safety Standard—Determination of Protein in Foods.

[19] (2016). National Food Safety Standard—Determination of Amino Acids in Foods.

[20] (2016). National Food Safety Standard—Determination of Fat in Foods.

[21] (2016). National Food Safety Standard—Determination of Fatty Acids in Foods.

[22] Li G.M., Liu L.P., Yin B., Liu Y.Y., Dong W.W., Gong S., Zhang J., Tan J.H. (2020). Heat stress decreases egg production of laying hens by inducing apoptosis of follicular cells via activating the FasL/Fas and TNF-alpha systems. Poult. Sci..

[23] Livak K.J., Schmittgen T.D. (2001). Analysis of relative gene expression data using real-time quantitative PCR and the 2^(−^*^ΔΔ^*^CT)^ method. Methods.

[24] Li Z., Zhu Q., Azad M.A.K., Li H., Huang P., Kong X. (2021). The impacts of dietary fermented Mao-tai lees on growth performance, plasma metabolites, and intestinal microbiota and metabolites of weaned piglets. Front. Microbiol..

[25] Qin B., Li Z., Zhu Q., Chen T., Lan W., Cui Y., Azad M.A.K., Kong X. (2024). Dietary fermented blueberry pomace supplementation improves small intestinal barrier function and modulates cecal microbiota in aged laying hens. Animals.

[26] Kowalczewski P.L., Olejnik A., Bialas W., Rybicka I., Zielinska-Dawidziak M., Siger A., Kubiak P., Lewandowicz G. (2019). The nutritional value and biological activity of concentrated protein fraction of potato juice. Nutrients.

[27] Hanlon C., Ziezold C.J., Bédécarrats G.Y. (2022). The diverse roles of 17β-estradiol in non-gonadal tissues and its consequential impact on reproduction in laying and broiler breeder hens. Front. Physiol..

[28] Santos R.D., Gagliardi A.C., Xavier H.T., Magnoni C.D., Cassani R., Lottenberg A.M., Arpadi F.A., Geloneze B., Scherr C., Kovacs C. (2013). First guidelines on fat consumption and cardiovascular health. Arq. Bras. Cardiol..

[29] Langley M.R., Triplet E.M., Scarisbrick I.A. (2020). Dietary influence on central nervous system myelin production, injury, and regeneration. Biochim. Biophys. Acta Mol. Basis Dis..

[30] Dedousi A., Kritsa M., Đukić Stojčić M., Sfetsas T., Sentas A., Sossidou E. (2022). Production performance, egg quality characteristics, fatty acid profile and health lipid indices of produced eggs, blood biochemical parameters and welfare indicators of laying hens fed dried olive pulp. Sustainability.

[31] Imamura F., Fretts A., Marklund M., Ardisson Korat A.V., Yang W., Lankinen M., Qureshi W., Helmer C., Chen T., Wong K. (2018). Fatty acid biomarkers of dairy fat consumption and incidence of type 2 diabetes: A pooled analysis of prospective cohort studies. PLoS Med..

[32] Jackson K.H., Harris W.S., Belury M.A., Kris-Etherton P.M., Calder P.C. (2024). Beneficial effects of linoleic acid on cardiometabolic health: An update. Lipids Health Dis..

[33] Omidi M., Rahimi S., Karimi Torshizi M.A. (2015). Modification of egg yolk fatty acids profile by using different oil sources. Vet. Res. Forum.

[34] Yan F., Zhao Q., Li Y., Zheng Z., Kong X., Shu C., Liu Y., Shi Y. (2022). The role of oxidative stress in ovarian aging: A review. J. Ovarian Res..

[35] Walzem R.L., Hansen R.J., Williams D.L., Hamilton R.L. (1999). Estrogen induction of VLDLy assembly in egg-laying hens. J. Nutr..

[36] Ma Y., Yao J., Zhou S., Mi Y., Tan X., Zhang C. (2020). Enhancing effect of FSH on follicular development through yolk formation and deposition in the low-yield laying chickens. Theriogenology.

[37] Samad N., Okonkwo C.E., Ayyash M., Al-Marzouqi A.H., Yuliarti O., Kamal-Eldin A. (2025). Valorization of fruit pomace by enzymatic treatment and microbial fermentation. Fermentation.

[38] Convissar S., Winston N.J., Fierro M.A., Scoccia H., Zamah A.M., Stocco C. (2019). Sp1 regulates steroidogenic genes and LHCGR expression in primary human luteinized granulosa cells. J. Steroid Biochem. Mol. Biol..

[39] Jarvensivu P., Saloniemi-Heinonen T., Awosanya M., Koskimies P., Saarinen N., Poutanen M. (2015). HSD17B1 expression enhances estrogen signaling stimulated by the low active estrone, evidenced by an estrogen responsive element-driven reporter gene in vivo. Chem. Biol. Interact..

[40] Wang X., Yin L., Geng C., Zhang J., Li J., Huang P., Li Y., Wang Q., Yang H. (2025). Impact of different feed intake levels on intestinal morphology and epithelial cell differentiation in piglets. J. Anim. Sci..

[41] Ensari A., Marsh M.N. (2018). Exploring the villus. Gastroenterol. Hepatol. Bed Bench.

[42] Feldman G.J., Mullin J.M., Ryan M.P. (2005). Occludin: Structure, function and regulation. Adv. Drug Deliv. Rev..

[43] Yu Y., Liu Z.Q., Liu X.Y., Yang L., Geng X.R., Yang G., Liu Z.G., Zheng P.Y., Yang P.C. (2013). Stress-derived corticotropin releasing factor breaches epithelial endotoxin tolerance. PLoS ONE.

[44] Shenoy S. (2019). CDH1 (E-Cadherin) mutation and gastric cancer: Genetics, molecular mechanisms and guidelines for management. Cancer Manag. Res..

[45] Turner J.R. (2009). Intestinal mucosal barrier function in health and disease. Nat. Rev. Immunol..

[46] Collins S.L., Stine J.G., Bisanz J.E., Okafor C.D., Patterson A.D. (2023). Bile acids and the gut microbiota: Metabolic interactions and impacts on disease. Nat. Rev. Microbiol..

[47] Stumpff F. (2018). A look at the smelly side of physiology: Transport of short chain fatty acids. Pflügers Archiv.–Eur. J. Physiol..

[48] Ballout J., Akiba Y., Kaunitz J.D., Diener M. (2021). Short-chain fatty acid receptors involved in epithelial acetylcholine release in rat caecum. Eur. J. Pharmacol..

[49] Naber E.C., Biggert M.D. (1989). Patterns of lipogenesis in laying hens fed a high fat diet containing safflower oil. J. Nutr..

[50] Zhao S., Jang C., Liu J., Uehara K., Gilbert M., Izzo L., Zeng X., Trefely S., Fernandez S., Carrer A. (2020). Dietary fructose feeds hepatic lipogenesis via microbiota-derived acetate. Nature.

[51] Rauckhorst A.J., Sheldon R.D., Pape D.J., Ahmed A., Falls-Hubert K.C., Merrill R.A., Brown R.F., Deshmukh K., Vallim T.A., Deja S. (2025). A hierarchical hepatic de novo lipogenesis substrate supply network utilizing pyruvate, acetate, and ketones. Cell Metab..

[52] Yi S., Zhang X., Zhang J., Ma Z., Wang R., Wu D., Wei Z., Tan Z., Zhang B., Wang M. (2022). Brittle Culm 15 mutation alters carbohydrate composition, degradation and methanogenesis of rice straw during in vitro ruminal fermentation. Front. Plant Sci..

[53] Ye L., Hou Y., Hu W., Wang H., Yang R., Zhang Q., Feng Q., Zheng X., Yao G., Hao H. (2023). Repressed *Blautia*-acetate immunological axis underlies breast cancer progression promoted by chronic stress. Nat. Commun..

[54] Chen H., Sun X., Huang H., Huang W., Liu C., Liu S. (2026). *Zongyangia acetica* sp. nov. and *Blautia acetica* sp. nov., two acetate-producing bacteria isolated from human fecal in the order Eubacteriales. Syst. Appl. Microbiol..

[55] Nogal A., Louca P., Zhang X., Wells P.M., Steves C.J., Spector T.D., Falchi M., Valdes A.M., Menni C. (2021). Circulating levels of the short-chain fatty acid acetate mediate the effect of the gut microbiome on visceral fat. Front. Microbiol..

[56] Yang R., Shan S., Shi J., Li H., An N., Li S., Cui K., Guo H., Li Z. (2023). *Coprococcus eutactus*, a potent probiotic, alleviates colitis via acetate-mediated IgA response and microbiota restoration. J. Agric. Food Chem..

[57] Zhou H., Huang D., Sun Z., Chen X. (2024). Effects of intestinal *Desulfovibrio* bacteria on host health and its potential regulatory strategies: A review. Microbiol. Res..

