# Supplementary material for: Dietary Fermented Strawberry Pomace Improves Yolk Lipid Quality and Modulates Reproductive Endocrine Activity, Intestinal Barrier Status, and Cecal Microbiota in Laying Hens
Source: Foods. 2026 Sep 20;15(18):3331. doi: 10.3390/foods15183331 (PMC13606238; doi:10.3390/foods15183331)
Supplement: Supplementary file 1 [file foods-15-03331-s001.zip › foods-4553479-supplementary.pdf]

Supplementary Table S1. Ingredients and nutrient contents of the basal diet (%, as feed basis).

**Ingredients**	**Content**	**Nutrients**[2]	**Level**
Corn	64.20	Metabolizable energy (MJ/kg)	11.38
Soybean meal	21.60	Crude protein	15.56
Limestone	8.00	Ether extract	5.10
Soybean oil	1.20	Crude ash	11.00
Premix[1]	5.00	Calcium	3.52
Total	100.00	Total phosphorus	0.42
		Methionine	0.33
		Lysine	0.95

[1]Premix supplied per kilogram of diet: Vitamin A, 10,000 IU; vitamin D_3, 3000 IU; vitamin K_3, 1.75 mg; vitamin E, 20 IU; vitamin B_1, 2 mg; vitamin B_2, 6 mg; vitamin B_6, 3 mg; vitamin B_{12}, 0.02 mg; pantothenic acid, 8.5 mg ; nicotinamide, 40 mg; folic acid, 1 mg; biotin, 0.24 mg; choline chloride, 450 mg; lysine, 0.95 g; methionine, 1.3 g; calcium, 5 g; phosphorus, 0.75 g; copper, 8 mg; Fe, 75 mg; Mn, 100 mg; Zn, 65 mg; I, 0.8 mg; Se, 0.3 mg; sodium chloride, 3 g.

[2] Metabolizable energy was calculated value, and other nutrient levels were measured values

Supplementary Table S2. Primer sequences used for quantitative real-time PCR analysis.

Gene	Accession number	Primer sequence (5′–3′)	Product size (bp)
β-actin	NM_205518.1	F:ATGAAGCCCAGAGCAAAAGA<br>R:GGGGTGTTGAAGGTCTCAAA	223
Cadherin 1	NM_001039258.3	F: TCGTCAGCTACTCCATCGTC<br>R: TCGTACATGGTGGGGTTGAA	247
CAT	NM_001031215.2	F: AGATGGCGTATGACCCTAGC<br>R: CCTCTGATAATTGGCCACGC	173
Claudin1	NM_001013611.2	F: AGGTGTACGACTCGCTGCTT<br>R: AGCCACTCTGTTGCCATACC	242
Claudin2	NM_001277622.1	F: CCTGCTCACCCTCATTGGAG<br>R: GCTGAACTCACTCTTGGGCT	144
CYP11A1	NM_001001756.2	F: CAGAGGCACAGCAGTTCATC<br>R:CACAAGGAGGCTGAAGAGGA	239
CYP17A1	NM_001001901.3	F: TGCTTCAACTCCTCCTACCG<br>R: CATCAGGTCCCTCACAGTGT	243
CYP19A1	NM_001001761.4	F: TGAGAGTTTGGATCAGCGGT<br>R:ACAAGACCAGGACCAGACAG	220
ESR1	NM_205183.2	F: AATGAACAGGGCAGCTTTGG	210

		R: CTCACAAGACCAGACCCCAT	
ESR2	NM_204794.3	F: CTCAGCACAGTCAGTCCAGA<br>R: AGACTTCCATCCAGCAGCTT	238
FSHR	NM_205079.2	F: GTAGCATGTCTCCGGCAAAG<br>R: GAAGGCATCAGGTTTGGGTG	185
GPX1	NM_001277853.2	F: ATGTTCGAGAAGTGCGAGGT<br>R: AGTTCCAGGAGACGTCGTTG	160
HO-1	NM_205344.1	F: ATGCCTACACCCGCTATTTG<br>R: ATCTCAAGGGCATTCATTCG	178
HSD17B1	NM_204837.1	F: GCAGTGTTTGAGGTGAACGT<br>R: CATGTGGATGTTGAAGGGCC	216
HSD3B1	NM_205118.2	F: GGCTGCTGGACAAAGACTTC<br>R: GCCCAAGGTGTCAATGATGG	173
Keap1	MN416132.1	F: CATCAACTGGGTGCAGTACG<br>R:AGGGTGAGGTCCTGGAAGAT	183
LHCGR	NM_204936.2	F: ATCTCCCAGAGTGACTCCCT<br>R: TCTGCGTCAAGTCTGGGAAT	193
Mucin 2	NM_001318434.1	F: TGTGTTTGAGAAGTGCCGTG<br>R: AGAGCAGCAAACACCATTGG	184
NQO1	NM_001277619.1	F: AAGAAGATTGAAGCGGCTGA<br>R: GCATGGCTTTCTTCTTCTGG	166
Nrf2	NM_205117.1	F: CCACCCTAAAGCTCCATTCA	217

		R: ATTCTTGCCTCTCCTGCGTA	
Occludin	NM_205128.1	F: CCGTAACCCCGAGTTGGAT<br>R: ATTGAGGCGGTCGTTGATG	214
SOD1	NM_205064.1	F: ATTACCGGCTTGTCTGATGG<br>R: CCTCCCTTTGCAGTCACATT	173
SOD2	NM_204211.2	F: CCTTCGCAAACTTCAAGGAG<br>R: CCAGCAATGGAATGAGACCT	162
ZO-1	XM_015278975.2	F: TTTACATGATGACCGCCTGT<br>R: GAATCCTCCCTAACGGGTTC	213

CAT, catalase; *CYP11A1*, cytochrome P450, family 11, subfamily A, polypeptide 1; *CYP17A1*, cytochrome P450, family 17, subfamily A, polypeptide 1; *CYP19A1*, cytochrome P450, family 19, subfamily A, polypeptide 1; *ESR*, estrogen receptor; *FSHR*, follicle-stimulating hormone receptor; *GPX1*, glutathione peroxidase 1; *HO-1*, heme oxygenase-1; *HSD17B1*, hydroxysteroid (17-beta) dehydrogenase 1; *HSD3B1*, hydroxysteroid (3-beta) dehydrogenase 1; *Keap1*, Kelch-like ECH-associated protein 1; *LHCGR*, luteinizing hormone/choriogonadotropin receptor; *NQO1*, NAD(P)H: quinone oxidoreductase 1; *Nrf2*, nuclear factor erythroid 2-related factor 2; *SOD*, superoxide dismutase; *ZO-1*, zonula occludens-1.

Supplementary Table S3. Ct values of β-actin in ovarian samples from different dietary treatments

Item	Control	Dietary FSP level (%)			SEM	*P*-values
		0.25	0.5	1.0		
β-actin（ovary）	17.22	17.03	16.81	16.72	0.242	0.467
β-actin（jejunum）	20.05	19.28	19.26	19.44	0.130	0.103
β-actin（ileum）	18.40	18.19	17.71	17.99	0.176	0.059

Supplementary Table S4. Effects of dietary fermented strawberry pomace supplementation on crude protein and amino acid composition of egg albumen in laying hens (g/100 g).

Item	Control	Dietary FSP level (%)			SEM	*P*-values
		0.25	0.5	1.0		
CP	9.93	9.48	10.16	9.91	0.104	0.128
Arginine	0.57	0.56	0.59	0.56	0.007	0.397
Histidine	0.23	0.22	0.24	0.23	0.003	0.226
Isoleucine	0.53	0.52	0.53	0.52	0.006	0.792
Leucine	0.86	0.84	0.87	0.85	0.009	0.637
Lysine	0.65	0.66	0.68	0.65	0.009	0.539
Methionine	0.49	0.49	0.49	0.52	0.011	0.833
Phenylalanine	0.62	0.60	0.62	0.60	0.007	0.494
Threonine	0.46	0.44	0.47	0.46	0.005	0.467
Valine	0.69	0.66	0.68	0.67	0.008	0.629
EAA	5.11	5.00	5.18	5.06	0.057	0.739
Alanine	0.61	0.58	0.61	0.59	0.007	0.348
Aspartic acid	1.06	1.00	1.05	1.03	0.011	0.269
Glutamic acid	1.37	1.31	1.38	1.34	0.016	0.441
Glycine	0.36	0.34	0.36	0.35	0.004	0.335
Proline	0.34^{a}	0.30^{b}	0.32^{ab}	0.30^{b}	0.004	0.004
Serine	0.70	0.65	0.69	0.68	0.009	0.165
Tyrosine	0.39	0.39	0.38	0.37	0.005	0.641
NEAA	4.83	4.57	4.79	4.67	0.053	0.334
FAA	3.97	3.78	3.98	3.87	0.043	0.354
BCAA	2.08	2.02	2.09	2.04	0.023	0.699
TAA	9.94	9.57	9.97	9.73	0.109	0.558

Data are expressed as means with their SEM (n = 6). ^{a,b} Mean values without a common superscript letter within the same row are differed significantly at $P < 0.05$. Essential amino acids include arginine, histidine, isoleucine, leucine, lysine, methionine, phenylalanine, threonine, valine, and proline. Non-essential amino acids include alanine, aspartic acid, glutamic acid, glycine, serine, and tyrosine. Flavor amino acids include aspartic acid, glutamic acid, glycine, alanine, and arginine. Branched-chain amino acids include isoleucine, leucine, and valine. CP, Crude protein; EAA, Essential amino acids; NEAA, Non-essential amino acids; FAA, Flavor amino acids; BCAA, Branched-chain amino acids; TAA, Total amino acids.
